# Blind recovery of sources for multivariate space-time random fields

**DOI:** 10.1007/s00477-022-02348-2

**Published:** 2022-12-30

**Authors:** C. Muehlmann, S. De Iaco, K. Nordhausen

**Affiliations:** 1 Institute of Statistics and Mathematical Methods in Economics, TU Wien / Technische Universität Wien / Vienna University of Technology, Vienna, Austria; 2 Department of Economic Sciences-Sect. of Mathematics and Statistics, University of Salento, Lecce, Italy; 3 Centro Nazionale di Biodiversità, University of Salento, Lecce, Italy; 4 Department of Mathematics and Statistics, University of Jyväskylä, Jyväskylä, Finland

**Keywords:** Latent random fields, Local covariance matrix, Unmixing matrix, Space-time multivariate data

## Abstract

With advances in modern worlds technology, huge datasets that show dependencies in space as well as in time occur frequently in practice. As an example, several monitoring stations at different geographical locations track hourly concentration measurements of a number of air pollutants for several years. Such a dataset contains thousands of multivariate observations, thus, proper statistical analysis needs to account for dependencies in space and time between and among the different monitored variables. To simplify the consequent multivariate spatio-temporal statistical analysis it might be of interest to detect linear transformations of the original observations that result in straightforward interpretative, spatio-temporally uncorrelated processes that are also highly likely to have a real physical meaning. Blind source separation (BSS) represents a statistical methodology which has the aim to recover so-called latent processes, that exactly meet the former requirements. BSS was already successfully used in sole temporal and sole spatial applications with great success, but, it was not yet introduced for the spatio-temporal case. In this contribution, a reasonable and innovative generalization of BSS for multivariate space-time random fields (stBSS), under second-order stationarity, is proposed, together with two space-time extensions of the well-known algorithms for multiple unknown signals extraction (stAMUSE) and the second-order blind identification (stSOBI) which solve the formulated problem. Furthermore, symmetry and separability properties of the model are elaborated and connections to the space-time linear model of coregionalization and to the classical principal component analysis are drawn. Finally, the usefulness of the new methods is shown in a thorough simulation study and on a real environmental application.

## Introduction

In environmental sciences, datasets are usually generated by tracking certain variables of interest at different spatial locations over time. Scientists measure soil features or pollutants at different monitoring points over a territory and throughout a certain span of time, as in De Iaco et al. (2019); Ding et al. (2021); Wang et al. (2021) to name some examples. The analysis of such spatio-temporal data needs to account for possible dependence in space and time for each measured variable but also in-between variables. This is usually achieved by assuming that the dataset is generated by a multivariate stochastic random field indexed in space and time, i.e.: $\{ \mathbf {x}(\mathbf {s}, t)=(x_1(\mathbf {s}, t), \dots , x_p(\mathbf {s}, t))^\top : (\mathbf {s}, t) \in \mathcal {S}\times \mathcal {T}\subseteq \mathbb {R}^{d + 1} \}$ where *p* is the dimension of ${ \mathbf {x}(\mathbf {s}, t)}$, $(\mathbf {s}, t)$ is a spatio-temporal location, $\mathcal {S}\subseteq \mathbb {R}^d$ is the spatial domain and $\mathcal {T}\subseteq \mathbb {R}$ is the temporal domain. Given the spatio-temporal random field $\mathbf {x}$, the corresponding first and second-order moments, assumed to be finite, are for all $({\mathbf {s}}, t), ({\mathbf {s}}', t') \in \mathcal {S}\times \mathcal {T}$

1 $$\begin{aligned}&\mathbb {E}({\mathbf {x}}({\mathbf {s}}, t)) =(\mathbb {E}(x_1(\mathbf {s}, t)), \dots , \mathbb {E}(x_p(\mathbf {s}, t)))^\top . \end{aligned}$$

2 $$\begin{aligned}&\mathop {\mathbf {Cov}}\nolimits ({\mathbf {x}}({\mathbf {s}}, t), {\mathbf {x}}({\mathbf {s}}', t')) = [C(x_i(\mathbf {s}, t), x_j({\mathbf {s}}', t'))]_{i,j=1,\ldots ,p}, \end{aligned}$$

where $$C(x_i(\mathbf {s}, t), x_j({\mathbf {s}}', t'))=\mathbb {E}(x_{i}(\mathbf {s}, t) x_{j}({\mathbf {s}}', t')) - \mathbb {E}(x_i(\mathbf {s}, t)) \mathbb {E}(x_j({\mathbf {s}}', t')).$$

Statistical modelling of these quantities is a challenging task as the mean is a *p*-dimensional vector-valued functional and the covariance is a $(p \times p)$ matrix-valued functional, both allowing a wide variety of spatio-temporal behaviors throughout the considered domain. To simplify this task one usually assumes that the stochastic random field is stationary in a weak sense, yielding that it has a constant mean over the whole spatio-temporal domain and that the covariance exhibits invariance under translation of the coordinates. These assumptions allow to focus on modelling the covariance which is only dependent on the separation vector between the spatio-temporal sample locations $({\mathbf {s}}, t)$ and $({\mathbf {s}}', t')$, i.e.,

3 $$\begin{aligned} \mathop {\mathbf {Cov}}\nolimits ({\mathbf {x}(\mathbf {s}, t)}, {\mathbf {x}}({\mathbf {s}}', t')) = \mathbf {C} (\mathbf {s}- \mathbf {s}', t - t') = \mathbf {C} (\mathbf {h}, \tau ), \end{aligned}$$

where $\mathbf {h}$ is the spatial lag vector and $\tau$ is the temporal lag. If additionally the covariance is only dependent on the norm of the spatial lag $h = \Vert \mathbf {h}\Vert$ then it is said to be (spatially) isotropic.

Similar to the sole spatial case, the extension from univariate to multivariate covariance modelling increases complexity and introduces further challenges as for example reviewed in Genton and Kleiber (2015). In its essence, the task of modeling a real valued covariance functional is made more difficult as now a full $(p \times p)$ matrix-valued functional needs to be modeled. One of the available approaches is based on building multivariate models from univariate space-time models. This approach was originally introduced in geostatistics and is known as linear model of coregionalization (LMC) (Journel and Huijbregts 1978; Goulard and Voltz 1992). Extensions of the LMC to spatio-temporal data (ST-LMC) have been already considered in the literature (De Iaco et al. 2003; Choi et al. 2009; De Iaco et al. 2013a). The ST-LMC is defined by

4 $$\begin{aligned} \mathbf {C}(\mathbf {h}, \tau ) = \sum ^r_{k=1} \mathbf {T}_k C_k(\mathbf {h}, \tau ), \end{aligned}$$

where $\mathbf {T}_k$ are positive-definite coregionalization matrices and $C_k(\mathbf {h}, \tau )$ are univariate (usually parametric) spatio-temporal covariance functions. Various classes of ST-LMCs can be obtained by choosing a specific model for the univariate covariance function $C_k(\mathbf {h}, \tau )$ among the families available in the literature. For example, in De Iaco et al. (2003); Choi et al. (2009) the basic structures were modeled through the use of the product-sum form (De Iaco et al. 2001b), whose expression is given by

5 $$\begin{aligned} C (\mathbf {h}, \tau ; k_1, k_2, k_3) = k_1 C^{t}(\tau ) C^{sp}(\mathbf {h}) + k_2 C^{sp}(\mathbf {h}) + k_3 C^{t}(\tau ), \end{aligned}$$

where $C^{t}(\tau )$ and $C^{sp}(h)$ are valid parametric covariance functions representing the temporal and spatial parts. $k_1 > 0, k_2 \ge 0,$ and $k_3 \ge 0$ are the weights, respectively, for the product, sole spatial and sole temporal part, ensuring the admissibility of the covariance model. This parametric spatio-temporal covariance model is constructed by considering the fact that products and sums of covariance models, defined on different factor spaces, are still admissible covariance models on a higher dimensional space (De Iaco et al. 2011). Moreover, it can describe the spatio-temporal behavior of a space-time random field which is formed by a product of two independent spatial and temporal non-zero mean random fields (Cappello et al. 2019). Note that the temporal part might be chosen as any covariance model originating from time series analysis, e.g.: an exponential form yielded by an autoregressive process of order one (AR(1)). In similar fashion, the spatial part might equal well-studied spatial covariance models such as the Matérn class (Guttorp and Gneiting 2006). It is worth to underline that given $k_2 = k_3 = 0$, the above form reduces to so-called separable covariance class. Another family of popular stationary univariate covariance models, introduced by Gneiting (2002), has the following form

6 $$\begin{aligned} C(\mathbf {h}, \tau ) = \sigma ^2 \psi (|\tau |^2)^{-d/2} \phi \left( \frac{ \Vert \mathbf {h}\Vert ^2}{\psi (|\tau |^2)} \right) , \end{aligned}$$

where $\psi (z), z\ge 0$, is a positive function with completely monotone derivative, $\phi (z), z\ge 0$, is a completely monotone function and $\sigma ^2$ is the variance parameter. As detailed in Gneiting (2002), this class is characterized by a space-time interaction parameter, however the functions $\psi$ and $\phi$ can be identified through the inspection of the temporal and the spatial marginal dependence structure of the random field. The above two classes of models are only two popular examples, however there is an abundance of other possibilities in the literature, a recent overview of models and general challenges regarding spatio-temporal modeling and testing is given by De Iaco et al. (2013b); Montero et al. (2015); Cappello et al. (2018); Porcu et al. (2021).

Closely related to the class of models in Eq. (4) is the fact that the covariance structure results from certain forms of random processes. In particular, the ST-LMC can be thought of as a linear-combination of $k=1,\dots ,r$ *p*-variate random processes where each entry of the *k*-th process has $C_k(\mathbf {h}, \tau )$ as spatio-temporal covariance function. These processes are often called latent or factors and the analyst is interested in estimating these as they often represent physical processes that act at different spatio-temporal scales. Generally, analysts often aim to find transformations of the data that reflect the underlying nature, for simplicity such transformations are often linear. A prominent example is the principal component analysis (PCA) (Jolliffe 1986) which is often applied to spatio-temporal data. Bauer-Marschallinger et al. (2013) analyze soil moisture data of Australia collected over space and time with PCA and find components that can be associated with certain climate modes as well as De Iaco et al. (2001a) who extract two total air pollution indicators from a multivariate space-time dataset. A general overview of the use of PCA for data with spatial and temporal dependencies is provided by Demsar et al. (2013). However, PCA is disadvantageous as its simplicity (orthogonal directions that maximize variance) leads to the issue that it does not account for spatio-temporal dependencies. Although there are efforts in the literature to introduce spatial or spatio-temporal information into the PCA methodology (Jombart et al. 2008; Harris et al. 2015) the drawbacks of orthogonal loadings and the fact that PCA does not rely on a specific statistical model still remain. A more sophisticated way of recovering meaningful latent processes is given by the framework of blind source separation (BSS) which is most known for the identically and independently distributed (iid) data where it is referred to as independent component analysis (ICA) (Comon and Jutten 2010). The simplest BSS model is based on the location-scatter model (Nordhausen and Oja 2018)

7 $$\begin{aligned} \mathbf {x} = \mathbf {A}\mathbf {z} + \mathbf {m}. \end{aligned}$$

In this model $\mathbf {x}$ and $\mathbf {z}$ are the *p*-variate observable and latent random vectors and the deterministic part is formed by $\mathbf {A}$, the $(p \times p)$ mixing matrix, and $\mathbf {m}$, the *p*-dimensional location vector. BSS aims to find a matrix that inverses the transformation given by $\mathbf {A}$ (and $\mathbf {m}$) and recover the latent process $\mathbf {z}$ which is meant to show the underlying physical processes of the data. BSS is used with great success on time series data as outlined by Pan et al. (2021) and it is also introduced for spatial data by Nordhausen et al. (2015). In both cases BSS is advantageous over the classical PCA for three reasons: (i) the interpretation of the BSS result follows the same simple but effective loadings-scores principle, (ii) the BSS loadings are not restricted to be orthogonal, and (iii) in contrast to PCA the BSS methods do not rely only on marginal second-order moments but on temporal/spatial dependencies specific to the structure of the data.

The combination of the framework of BSS with the space-time geostatistical approach provides tools for data exploration that extract meaningful and interpretative features of the space-time data at hand. This is very useful in a variety of applications, such as the ones in the environmental and Earth sciences, meteorology, hydrology, since multivariate modeling problems in space-time can be treated as several univariate spatio-temporal issues with a remarkable simplification from a computational point of view. In particular, the novelty of this paper can be recognized especially in the generalization of BSS for multivariate space-time random fields (stBSS) and in the two space-time extensions of the algorithms for multiple unknown signals extraction (stAMUSE) and second-order blind identification (stSOBI), where specific theoretical and practical aspects related to a joint space-time context are discussed. In addition, the concepts of symmetry and separability of the model are introduced and the connections to the space-time linear model of coregionalization and to the classical principal component analysis are drawn. It is also worth pointing out that the proposed generalization diverges from the existing BSS methods for space-time data (Douglas et al. 2007; Ashino et al. 2009; de Jesús Nuño Ayón et al. 2018). All these methods work in a very different setting with respect to the one considered hereafter. Indeed, in the signal processing community, where BSS originates, the components of $\mathbf {z}$, in Model (7), correspond to sensors placed at different spatial locations which are observed over time, and therefore they refer then to the dependence between the components of $\mathbf {z}$ as spatial dependence. Coordinate information is usually not available or not used and actually often the goal is to locate a sensor. Thus, spatio-temporal BSS methods developed in such a context, are not applicable to the framework considered in this paper, where it is assumed to have *p* measurements observed over time at a given location. In other terms, in the subsequent formulation known sample locations are assumed, the source random fields exhibit space-time dependence and the mixture is instantaneous. These assumptions are well suited in space-time geostatistical applications, as also highlighted in a simulation study and in a real environmental analysis.

The structure of this paper is outlined as follows. Section 2 details the spatio-temporal BSS model and puts it into the perspective of the statistical analysis of spatio-temporal data by considering properties such as separability, symmetry and the connection to other spatio-temporal models. Section 3 introduces estimators for the unmixing matrix and the latent process, shows identifiability and affine equivalence properties and connects the BSS methods to the classical PCA. The usefulness of these introduced estimators is shown in Sect. 4 by a thorough simulation study and in Sect. 5 on a real dataset. Section 6 concludes and outlines possible further research.

## Spatio-temporal blind source separation model

Based on spatial BSS (SBSS) originally introduced and studied by Nordhausen et al. (2015); Bachoc et al. (2020), an extension to the space-time setting can be achieved by considering a random field ${\mathbf {x}}$ defined on a higher dimensional domain $\mathbb {R}^{d + 1}$, where *d* is the dimension of the spatial domain and the additional dimension is for time. However, this generalization from space to space-time is not straightforward, since some specific theoretical and practical issues have to be faced, such as the ones related to metric, sampling and peculiar characteristics of the data. First of all, the definition of a metric that combines the spatial and temporal dimensions is challenging as the physical units of space and time are not comparable, thus, constants that either cast space to time units (or vice-versa) need to be introduced. To overcome this issue the spatial and temporal coordinates can be kept separate. In addition, regarding the space-time data sampling, it is typical to have sparse sample locations in space, but dense in time. This can be often found in environmental monitoring systems, where only a relatively low number of survey stations are available due to the relatively high cost of necessary measurement equipment and the limited accessibility over the area. In contrast, measuring stations commonly take measurements continuously over long periods of time resulting in regular high resolution time series. Hence, the most common data sets in a space-time context are sparse in space and dense in time. As a consequence, the spatial and temporal correlations are estimated with different degrees of reliability and accuracy. As a third point, space and time have their fundamental peculiarities since there is a natural order in time (i.e.: past, present and future), while for space, there is generally no ordering, but it is of interest focusing on potential directional dependence, known as anisotropy. Thus, these aspects can influence the construction of the local autocovariance matrices and the spatio-temporal kernel, where the concepts of full symmetry and separability, well-known in space-time geostatistics, should be also considered.

In the light of the former aspects, the definition of a stationary spatio-temporal BSS (stBSS) model is introduced as follows.

### Definition 2.1

Given a multivariate random field $\{{\mathbf {x}(\mathbf {s}, t)}, ({\mathbf {s}}, t)\in \mathcal {S}\times \mathcal {T}\}$, with *p* components defined on the spatio-temporal domain $\mathcal {S}\times \mathcal {T}\subseteq \mathbb {R}^{d+1}$ of dimension $d+1$, a stationary spatio-temporal blind source separation (stBSS) model is such that

8 $$\begin{aligned} \mathbf {x}(\mathbf {s}, t)= \mathbf {A}\mathbf {z}(\mathbf {s}, t)+ \mathbf {m}, \end{aligned}$$

where the *p*-variate random field $\mathbf {z}(\mathbf {s}, t)$ consists of second-order stationary and uncorrelated components ${z}_i({\mathbf {s}},t)$, $i=1, \dots , p$ with zero expected value and unit variance, $\mathbf {A}$ is the deterministic mixing matrix of full-rank of dimension $(p\times p)$, $\mathbf {m}$ is a *p*-dimensional deterministic location vector.

Equation (8) represents the location scatter model for spatio-temporal random fields (more details of the location scatter model are provided by Nordhausen and Oja (2018)). Note that the random field $\mathbf {x}(\mathbf {s}, t)$ is said observable, since a finite realization of $\mathbf {x}(\mathbf {s}, t)$ is assumed available, the *p*-variate random field $\mathbf {z}(\mathbf {s}, t)$ is said latent. The assumptions on the latent field in the former definition translate to the following ones in more mathematical terms. For any pair of spatio-temporal locations $(\mathbf {s},t), (\mathbf {s}',t') \in \mathcal {S}\times \mathcal {T}$, separated by the spatio-temporal lag $(\mathbf {h}, \tau ) = (\mathbf {s}- \mathbf {s}', t - t')$, the latent field $\mathbf{z}(\mathbf{s},t)$ fulfills the following two conditions:

(stBSS 1): $\mathbb {E}( \mathbf {z}(\mathbf {s}, t)) = \mathbf{0}$, $\mathop {\mathbf {Cov}}\nolimits (\mathbf {z}(\mathbf {s}, t))=\mathbf {I}_p$, where $\mathbf{I}_p$ is the identity matrix of order *p*,
(stBSS 2): $\mathop {\mathbf {Cov}}\nolimits ({\mathbf {z}(\mathbf {s}, t)}, {\mathbf {z}}({\mathbf {s}}', t')) = \mathbb {E}({\mathbf {z}(\mathbf {s}, t)} {\mathbf {z}}({\mathbf {s}}', t')) = {\mathbf {D}}({\mathbf {h}},\tau )$, where ${\mathbf {D}}({\mathbf {h}}, \tau )$ is a diagonal matrix, whose *i*-th diagonal element is the covariance of the *i*-th entry of the latent process $\mathbf {z}(\mathbf {s}, t)$. That is ${\mathbf {D}}_i({\mathbf {h}}, \tau ) = C_{i}({\mathbf {h}}, \tau )$.

Condition (stBSS 1) reflects the zero mean and unit covariance condition which is usually stated in BSS as it ensures an identifiable location vector and a more identifiable mixing matrix as will be discussed in detail in Sect. 3. The latter condition is specific to the stBSS model as it states the second-order stationary property of the latent field. Still, both conditions do not specify the model completely as for a given pair of $(\mathbf {A}, \mathbf {z}(\mathbf {s}, t))$, the pair $(\mathbf {A}\mathbf {P} \mathbf {J}, \mathbf {J} \mathbf {P}^\top \mathbf {z}(\mathbf {s}, t))$ leads to the same observable $\mathbf {x}(\mathbf {s}, t)$ where the two latent fields $\mathbf {z}(\mathbf {s}, t)$ and $\mathbf {J} \mathbf {P}^\top \mathbf {z}(\mathbf {s}, t)$ do not violate conditions (stBSS 1) and (stBSS 2). This holds true for any $\mathbf {P} \in \mathcal {P}^p$ and $\mathbf {J} \in \mathcal {J}^p$ where $\mathcal {P}^p$ is the set of all $(p \times p)$ permutation matrices and $\mathcal {J}^p$ is the set of all sign-change matrices, i.e., diagonal matrices with diagonal elements $\pm 1$. Hence, the latent field (or equivalently the mixing matrix) is only identifiable up to permutation and sign (of its rows) which is generally the case in BSS and not considered as a problem. In order to ensure parameter identifiability, conditions (stBSS 2) needs to be replaced by a slightly stricter assumption depending on the used estimator as discussed in detail in Sect. 3.

Conditions (stBSS 1) and (stBSS 2) determine the second-order dependence of the observable field. For any couple of spatio-temporal locations $(\mathbf {s},t), (\mathbf {s}',t') \in \mathcal {S}\times \mathcal {T}$, separated by the lag $(\mathbf {h}, \tau ) = (\mathbf {s}- \mathbf {s}', t - t')$, to be

9 $$\begin{aligned} \mathop {\mathbf {Cov}}\nolimits (\mathbf {x}(\mathbf {s}, t)) = \mathbf {A}\mathbf {A}^\top \quad \text {and} \quad \mathop {\mathbf {Cov}}\nolimits (\mathbf {x}(\mathbf {s}, t), \mathbf {x}(\mathbf {s}', t')) = \mathbf {A}{\mathbf {D}}({\mathbf {h}}, \tau ) \mathbf {A}^\top . \end{aligned}$$

This relation hints the advantage of modeling the observable with a stBSS model as the second-order dependence is completely defined by a $p \times p$ matrix and *p* univariate stationary spatio-temporal covariance functions in contrast to *p* covariance and $p(p-1)/2$ cross-covariance functions when no assumptions on $\mathbf {x}(\mathbf {s}, t)$ are stated. In this view, stBSS simplifies the second-order spatio-temporal dependence drastically. In the following, more properties of the stBSS model related to spatio-temporal second-order statistics are outlined.

Connection to the LMC The stBSS model represents a special case of the ST-LMC as the ST-LMC from Eq. (9) can be rewritten under the stBSS model as

10 $$\begin{aligned} \mathop {\mathbf {Cov}}\nolimits (\mathbf {x}(\mathbf {s}, t), \mathbf {x}(\mathbf {s}+ \mathbf {h},t + \tau )) = \sum _{i=1}^p \mathbf {a}_i \mathbf {a}_i^\top C_i (\mathbf {h}, \tau ), \end{aligned}$$

where $\mathbf {a}_i$ are the column vectors of the mixing matrix $\mathbf {A}$; thus this is a special form of a ST-LMC (Eq. (4)) defined by *p* basic covariance structures $C_i$ associated to the entries of the latent process $\mathbf {z}(\mathbf {s}, t)$ and the rank-one positive semi-definite coregionalization matrices $\mathbf {a}_i \mathbf {a}_i^\top$. It is worth emphasizing that the stBSS methodology has significant advantages over the multivariate geostatistical analysis based on the ST-LMC. Firstly, stBSS is not meant to only focus on modelling of the covariance function of the data. It is a framework that decomposes multivariate dependent observations into a set of of *p* univariate uncorrelated or independent components which allows to model each component individually discarding complex multivariate modelling as discussed above. Secondly, estimation of the latent process does only rely on the mild assumptions on the latent process stated above and estimating the covariance functions of the entries of the latent process is completely avoided. Lastly, as aslo outlined in Sect. 3, the unmixing matrix functionals show properties of high practical relevance, e.g.: affine equivariance which makes the estimation independent of the actual way of mixing.

Symmetry Full symmetry of the spatio-temporal covariance function is attained if it holds that

11 $$\begin{gathered} {\mathbf{Cov}}_{{\mathbf{x}}} ({\mathbf{h}},\tau ) = {\mathbf{Cov}}_{{\mathbf{x}}} ( - {\mathbf{h}}, - \tau )\quad {\text{and}} \hfill \\ {\mathbf{Cov}}_{{\mathbf{x}}} ({\mathbf{h}},\tau ) = {\mathbf{Cov}}_{{\mathbf{x}}} ( - {\mathbf{h}},\tau ) = {\mathbf{Cov}}_{{\mathbf{x}}} ({\mathbf{h}}, - \tau ). \hfill \\ \end{gathered}$$

Further inspection of Eq. 10 indicates that for the observable random field the left part of Eq. 11 is fulfilled because univariate covariance functions are symmetric, i.e.: $C_i (\mathbf {h}, \tau ) = C_i (-\mathbf {h}, -\tau )$ for all $i=1,\dots ,p$. Indeed, the hypothesis of symmetry represents in general an assumption of the ST-LMC. In addition, a sufficient condition that the right part of Eq. 11 holds is that all univariate covariance functions are fully symmetric, yielding for all $i=1,\dots ,p$ that $C_i (\mathbf {h}, \tau ) = C_i (-\mathbf {h}, \tau )$.

Separability Under separability, the covariance function related to each element of $\mathbf {z}(\mathbf {s}, t)$ is formed by a product of a sole spatial and a sole temporal part, i.e.: $C_{i}(\mathbf{h},\tau ) = C_i^{sp} (\mathbf {h}) C_i^{t}(\tau )$. This emerges when the latent field is formed by an elementwise product $\mathbf {z}(\mathbf {s}, t)= \mathbf {z}(\mathbf {s}) \circ \mathbf {z}(t)$ where the two factors $\mathbf {z}(\mathbf {s}) = ( z_1(\mathbf {s}), \dots , z_p(\mathbf {s}))^\top$ and $\mathbf {z}(t) = ( z_1(t), \dots , z_p(t))^\top$ are independent and are associated to the pure spatial and the pure temporal part, respectively, here $\circ$ denotes the Hadamard product. In matrix notation this yields ${\mathbf {D}}({\mathbf {h}}, \tau ) = {\mathbf {D}}^{sp}({\mathbf {h}}) \circ {\mathbf {D}}^t(\tau )$ where ${\mathbf {D}}^{sp}({\mathbf {h}})$ and ${\mathbf {D}}^t(\tau )$ are diagonal matrices holding the corresponding spatial and temporal univariate covariance functions $C_i^{sp} (\mathbf {h})$ and $C_i^{t}(\tau )$ as their diagonal elements. This is in a similar context denoted as space-time *p*-separable by Alegria et al. (2019). Note, that if the latent field is space-time *p*-separable then it is also fully symmetric as ${\mathbf {D}}({\mathbf {h}}, \tau ) = {\mathbf {D}}^{sp}({\mathbf {h}}) \circ {\mathbf {D}}^t(\tau ) = {\mathbf {D}}^{sp}({-\mathbf {h}}) \circ {\mathbf {D}}^t(\tau ) = {\mathbf {D}}({-\mathbf {h}}, \tau )$, the converse is not true. If the unit covariance condition $C_i^{sp} (\mathbf {0}) = C_i^{t}(0) = 1$ is utilized, it turns out that the marginal spatio-temporal covariance functions of $\mathbf {x}$, For any couple of spatio-temporal locations $(\mathbf {s},t), (\mathbf {s}',t') \in \mathcal {S}\times \mathcal {T}$, separated by the lag $(\mathbf {h}, \tau ) = (\mathbf {s}- \mathbf {s}', t - t')$, are defined as follows

12 $$\begin{aligned} \mathop {\mathbf {Cov}}\nolimits (\mathbf {x}(\mathbf {s}, t), \mathbf {x}(\mathbf {s},t + \tau )) = \mathbf {A}{\mathbf {D}}^t(\tau ) \mathbf {A}^\top \quad \text {and} \quad \mathop {\mathbf {Cov}}\nolimits (\mathbf {x}(\mathbf {s}, t), \mathbf {x}(\mathbf {s}+ \mathbf {h},t)) = \mathbf {A}{\mathbf {D}}^{sp}({\mathbf {h}}) \mathbf {A}^\top . \end{aligned}$$

The two models above are a sole spatial (left equation) and a sole temporal (right equation) BSS models with equal mixing matrices. Equivalently they can be interpreted as two ST-LMCs with equal rank-one coregionalization matrices but different (univariate) second-order dependencies. As outlined for the ST-LMC by De Iaco et al. (2003), this leads to the fact that analyzing the marginal temporal or spatial dependence (${\mathbf {D}}({\mathbf {0}}, \tau )$ or ${\mathbf {D}}({\mathbf {h}}, 0)$) is equivalent of analyzing the basic structures ${\mathbf {D}}^t(\tau )$ or ${\mathbf {D}}^{sp}({\mathbf {h}})$. It can also be seen from the right part of Eq. (9) that a space-time *p*-separable latent field translates to a space-time *p*-separable observable if $\mathbf {A}$ equals the trivial mixing, i.e.: $\mathbf {A}= \mathbf {P} \mathbf {J} \mathbf {D}$ for any $\mathbf {P} \in \mathcal {P}^p$, $\mathbf {J} \in \mathcal {J}^p$ and a diagonal matrix with non-negative diagonal entries $\mathbf {D}$.

## Unmixing matrix functionals

On the basis of the model introduced before (Definition 2.1), stBSS is concerned with recovering the latent process $\mathbf {z}(\mathbf {s}, t)$ by $\mathbf {W}(\mathbf {x}(\mathbf {s}, t)) (\mathbf {x}(\mathbf {s}, t)- \mathbf {T}(\mathbf {x}(\mathbf {s}, t)))$. Here, $\mathbf {W}$ is the so-called unmixing matrix and $\mathbf {T}(\mathbf {x}(\mathbf {s}, t))$ is a location vector functional. The following discussion is solely focused on $\mathbf {W}$. For $\mathbf {T}$ any location functional can be considered, usually, the standard sample mean is used.

### General properties of unmixing matrix functionals

Generally, in the BSS literature proper unmixing matrix functionals are assumed to be identifiable in the sense that $\mathbf {W}$ equals $\mathbf {A}^{-1}$ up to the model indeterminacies (order and sign) and they need to be affine equivariant. Both properties are usually formalized in the context of the considered model, e.g.: for iid data see Miettinen et al. (2015), for time series data see Matilainen et al. (2015) or for non-stationary spatial data see Muehlmann et al. (2022a). The following definition formalizes these desired properties for the stBSS case.

#### Definition 3.1

Let $\mathbf {x}(\mathbf {s}, t)$ be a *p*-variate random field originating from the stBSS model (Definition 2.1). An unmixing matrix functional $\mathbf {W}= \mathbf {W}(\mathbf {x}(\mathbf {s}, t))$, of dimension $(p\times p)$, is said to be valid if it possesses the following properties.

(Identifiability): $\mathbf {W}(\mathbf {z}(\mathbf {s}, t)) = \mathbf {P} \mathbf {J}$ for any $\mathbf {P} \in \mathcal {P}^p$ and $\mathbf {J} \in \mathcal {J}^p$.
(Affine equivariance): $\mathbf {W}(\mathbf {M}\mathbf {x}(\mathbf {s}, t)+ \mathbf {a}) = \mathbf {P} \mathbf {J} \mathbf {W}(\mathbf {x}(\mathbf {s}, t)) \mathbf {M} ^{-1}$ for any invertible matrix $\mathbf {M}$ of dimension $(p \times p)$, any vector $\mathbf {a}$ of dimension *p*, any $\mathbf {P} \in \mathcal {P}^p$ and $\mathbf {J} \in \mathcal {J}^p$.

The affine equivariance property reflects the motivation of BSS. Namely, physical processes (the latent processes) are measured by sensor and the sensor placement defines the way of mixing (mixing matrices). As physical processes act independently of the way of measuring they also should be recovered independently of the way of mixing. Hence, the signal recovery is desired to possess the affine equivariance property. This property is also useful when linear transformations are carried out prior the actual BSS analysis, which occurs in the analysis of data where the relevant information is found in relative values denoted as compositional data (Nordhausen et al. 2015).

Finding an unmixing matrix that reverses the location scatter model is a demanding task as the unmixing matrix (or equivalently the mixing matrix) are only restricted to be of full rank. Efforts have been made in the context of BSS to simplify this task. Usually, this is done by whitening and then finding an orthogonal matrix which is suggested by the following result.

#### Lemma 3.1

Given be a *p*-variate random field $\mathbf {x}(\mathbf {s}, t)$ originating from the stBSS model (Definition 2.1), let $\mathbb {E}[\mathbf {x}(\mathbf {s}, t)] = \mathbf {m}$ be the mean vector and $\mathop {\mathbf {Cov}}\nolimits (\mathbf {x}(\mathbf {s}, t)) = \mathbf {A}\mathbf {A}^\top$ the covariance matrix. Whitening the observable process yields $\mathbf {x}^{wh} (\mathbf {s},t) = \mathop {\mathbf {Cov}}\nolimits ^{-1/2}(\mathbf {x}(\mathbf {s}, t)) \left( \mathbf {x}(\mathbf {s}, t)- \mathbf {m} \right)$. Then, it holds that

13 $$\begin{aligned} \mathbf {x}^{wh} (\mathbf {s},t) = {\mathbf {U}}^\top \mathbf {z}(\mathbf {s}, t), \end{aligned}$$

denoting with ${\mathbf {U}}$ an orthogonal matrix of order *p*.

For the proof of Lemma 3.1 readers are referred to (Miettinen et al. (2015), Theorem 1). Based on this result an unmixing matrix functional can be defined by a three step outline: (i) the observable is whitened with respect to the covariance $\mathop {\mathbf {Cov}}\nolimits (\mathbf {x}(\mathbf {s}, t))$, (ii) an orthogonal matrix ${\mathbf {U}}$ is found that maximizes suitable information criteria as outlined below, and (iii) the former two steps are combined which yields $\mathbf {W}= {\mathbf {U}}\mathop {\mathbf {Cov}}\nolimits ^{-1/2}(\mathbf {x}(\mathbf {s}, t))$. In the literature, BSS can be roughly grouped by the way of finding the orthogonal matrix in Step (ii). One way is based on projection pursuit ideas and originally introduced in the context of ICA, denoted as FastICA (Nordhausen and Oja 2018). Another approach is followed by so-called algebraic BSS methods which state assumptions on moments in the considered model and find ${\mathbf {U}}$ by simultaneous or joint diagonalization of matrix functionals based on moments. For example, the algorithm for multiple unknown signals extraction (AMUSE) (Tong et al. 1990) or the second-order blind identification (SOBI) (Belouchrani et al. 1997) are designed for stationary time series and find ${\mathbf {U}}$ by diagonalizing one or more autocovariance matrices. In similar fashion SBSS extends this concept by using local covariance matrices which measure spatial second-order dependence and diagonalizes these quantities. In the subsequent section, these former approaches have been combined in order to provide a methodology to find an unmixing matrix for stationary spatio-temporal data.

### Two stBSS unmixing matrix functionals

As already clarified, the spatio-temporal covariance function of the latent random field $\mathbf {z}$ is the diagonal matrix ${\mathbf {D}}({\mathbf {h}}, \tau )$. Consequently, it is convenient to adopt the strategy of algebraic BSS methods and diagonalize scatter matrices that measure spatio-temporal second order dependence evaluated on the whitened version of the observable random process. Thus, the definition of population local covariance (or scatter) matrices (Bachoc et al. 2020; Muehlmann et al. 2020) is generalized to the space-time domain as follows. From now on it is assumed that the observable process is observed on a set of *n* spatio-temporal sample locations $\{ (\mathbf {s}_i, t_i) : i = 1, \dots , n, (\mathbf {s}_i, t_i) \in \mathcal {S}\times \mathcal {T}\}$. Local autocovariance matrices (LACF) are defined by

14 $$\begin{aligned} \mathop {\mathbf {LACF}}\nolimits _f(\mathbf {x}(\mathbf {s}, t)) = \frac{1}{n F_{n,f}} \sum _{i,j=1}^n f(\mathbf{s}_i - \mathbf{s}_j, t_i - t_j) \mathbb {E}\left( ({\mathbf {x}}({\mathbf {s}}_i, t_i) - \mathbb {E}(\mathbf{x}(\mathbf{s}_i, t_i))) (\mathbf{x}(\mathbf{s}_j, t_j) - \mathbb {E}(\mathbf{x}(\mathbf{s}_j, t_j))) ^ \top \right) \end{aligned}$$

with

15 $$\begin{aligned} F^2_{n,f} = \frac{1}{n}\sum _{i,j=1}^n f^2(\mathbf{s}_i - \mathbf{s}_j, t_i - t_j), \end{aligned}$$

where $f : \mathbb {R}^{d + 1} \rightarrow \mathbb {R}$ represents the spatio-temporal kernel function, which generates the weights. The normalization constant $F_{n,f}$ might be viewed as the average number of neighbouring spatio-temporal locations considered by the kernel function *f*.

The corresponding sample version of local autocovariance matrices is given by

16 $$\begin{aligned} \widehat{\mathop {\mathbf {LACF}}\nolimits }_f(\mathbf {x}(\mathbf {s}, t)) = \frac{1}{n F_{n,f}} \sum _{i,j=1}^n f(\mathbf{s}_i - \mathbf{s}_j, t_i - t_j) ({\mathbf {x}}({\mathbf {s}}_i, t_i) - \bar{\mathbf{x}}) (\mathbf{x}(\mathbf{s}_j, t_j) - \bar{\mathbf{x}})^\top , \end{aligned}$$

where $\bar{\mathbf{x}}$ is the sample mean vector. It is evident that local autocovariance matrices compute a weighted average of covariance matrices between all $n^2$ possible pairs of spatio-temporal sample locations. However, the weights, determined by the spatio-temporal kernel functions *f*, might be of different forms and might depend on the decision to introduce a metric into the higher dimensional space or to keep the spatial and temporal coordinates separate. In the former case, it would be necessary to define a specific distance function $h_{st}$, where space and time are combined through the use of positive real coefficient $a_1$ and $a_2$ which enable the comparison between disparate units of measure (such as meters and hours), that is:

17 $$\begin{aligned} h_{st}=(a_1 \Vert \mathbf{h}\Vert ^2+a_2|\tau |^2)^{0.5}, \end{aligned}$$

where $\Vert \mathbf{h}\Vert$ represents the Euclidean distance in space and $|\tau |$ the temporal lag. Thus, the kernels can be fixed as functions of the above distance; on the other hand, the kernel functions can be defined in such a way that a metric in space-time is not required, taking into account that space and time cannot be directly comparable, as specified below:

- Ball kernel: $f_b(\mathbf{{h}},\tau ;r_s,r_t) = I(\Vert \mathbf{{h}}\Vert \le r_s)I(|\tau |\le r_t)$, with $r_s\ge 0,r_t\ge 0$ and *I* the indicator function,
- Ring kernel: $f_r(\mathbf{{h}},\tau ;r_{s_0},r_{s_1},r_{t_0},r_{t_1})=I(r_{s_1}<\Vert \mathbf{h}\Vert \le r_{s_0})I( r_{t_1}<|\tau |\le r_{t_0})$, with $r_{s_0}>r_{s_1}\ge 0, r_{t_0}>r_{t_1}\ge 0$ and *I* the indicator function,
- Gauss kernel: $f_g(\mathbf{{h}},\tau ;r_s,r_t)=exp(-0.5\Phi ^{-1}(0.95)^2 [(\Vert \mathbf{h}\Vert / r_s)^2+(|\tau |/ r_t)^2])$, where $\Phi ^{-1}(0.95)$ is the 95% quantile of the standard Normal distribution and $r_s\ge 0,r_t\ge 0$.

Note that in the above case the concept of isotropy, which has in general no meaning for spatio-temporal random fields, is not recalled, and the assumption of anisotropy is used instead. It is worth pointing out that the Gauss kernel is the only function which can be separable and isotropic (De Iaco et al. 2020), thus defining a spatio-temporal metric or keeping separate the two distances is essentially equivalent. In the following, for a given second order stationary space-time random process, it is assumed that it is spatially isotropic in the weak sense (alternatively called second order isotropic), that is the covariance is a function of the spatial and temporal distances $\Vert \mathbf {h}\Vert$ and $|\tau |$, respectively. If the temporal part of the data is given in equidistant times the ring and ball kernel might be adapted in the sense that the temporal indicator function is replaced by an indicator function which captures certain lags, i.e.: $I(|\tau | = r_t)$.

For the whitening step, the covariance matrix can be expressed as a local autocovariance matrix by using a ball kernel function with a parameter $r_s = r_t = 0$, or $f=f_0=f_b(\cdot ;0,0)$, that is

18 $$\begin{aligned} \mathop {\mathbf {LACF}}\nolimits _{f_0}(\mathbf {x}(\mathbf {s}, t)) = n^{-1} \sum _{i=1}^n \mathbb {E}\left( ({\mathbf {x}}({\mathbf {s}}_i, t_i) - \mathbb {E}(\mathbf{x}(\mathbf{s}_i, t_i))) (\mathbf{x}(\mathbf{s}_i, t_i) - \mathbb {E}(\mathbf{x}(\mathbf{s}_i, t_i))) ^ \top \right) , \end{aligned}$$

and similarly the estimator of ${\mathop {\mathbf {LACF}}\nolimits }_{f_0}$ can be obtained from Eq. (16).

As in the spatial case, $\mathop {\mathbf {LACF}}\nolimits _{f_0}(\mathbf {x}(\mathbf {s}, t))$ can be considered a proper choice for whitening and $\mathop {\mathbf {LACF}}\nolimits _{f}(\mathbf {x}(\mathbf {s}, t))$ matrices are diagonal for the latent random process of a given stBSS model. Hence, the stBSS unmixing matrix functionals can be defined based on simultaneous/joint diagonalization as typically used in algebraic BSS methods as follows.

#### Definition 3.2

Given a *p*-variate random process $\mathbf {x}(\mathbf {s}, t)$, for which the stBSS model holds, as in Definition 2.1, let *f* be a spatio-temporal kernel function. The stAMUSE unmixing matrix functional ${\mathbf {W}} = {\mathbf {W}}(\mathbf {x}(\mathbf {s}, t))$ is based on simultaneous diagonalization and satisfies

19 $$\begin{aligned} \mathbf {W}\mathop {\mathbf {LACF}}\nolimits _{f_0}(\mathbf {x}(\mathbf {s}, t)) \mathbf {W}^\top = {\mathbf {I}}_p \qquad \text {and} \qquad \mathbf {W}\mathop {\mathbf {LACF}}\nolimits _{f}(\mathbf {x}(\mathbf {s}, t)) \mathbf {W}^\top = {\mathbf {D}}_f, \end{aligned}$$

where $\mathbf{D}_f$ is a diagonal matrix where its diagonal entries are ordered decreasingly.

The above spatio-temporal unmixing matrix functional can be seen as a direct extension of the AMUSE matrix (Tong et al. 1990) as the covariance and one local autocovariance matrix are simultaneously diagonalized, hence it is referred to as spatio-temporal AMUSE (stAMUSE). Similarly, it can be interpreted as a generalization of the SBSS (Nordhausen et al. 2015; Bachoc et al. 2020) methods, when only one local covariance matrix is used, instead of more than one as in Bachoc et al. (2020). Exact simultaneous diagonalization of the two involved quantities for a given sample is always possible by solving the generalized eigenvalue problem, see for example (Harville (1997), Chapter 21). If the diagonal elements of $\mathop {\mathbf {LACF}}\nolimits _{f}(\mathbf {z}(\mathbf {s}, t))$ are pairwise distinct, then the solution is unique up to sign (the order of the diagonal elements of $\mathbf{D}_f$ fixes the order and the scale is fixed by the stBSS model). Moreover, this method is affine equivariant. Both statements are formalized in the subsequent proposition.

#### Proposition 3.1

The following two properties hold for the stAMUSE functional (Definition 3.2):

1. Identifiable iff all diagonal elements of matrix $\mathop {\mathbf {LACF}}\nolimits _{f}(\mathbf {z}(\mathbf {s}, t))$ (which is itself a diagonal matrix) are pairwise distinct and
2. Affine equivariant.

Note that this method can be viewed as a particular case of the one introduced below, then Proposition 3.1 can be derived from Proposition 3.2, hence, the proof follows in the same manner as outlined below. This identifiability condition is a joint property of the actual covariance functions for each entry of $\mathbf {z}(\mathbf {s}, t)$ as well as of the used spatio-temporal kernel function which leads to the fact that the type of the kernel function is critical for the performance of this method. However, in order to over come this strong dependency, it might be useful to jointly diagonalize several local autocovariance matrices with different spatio-temporal kernel functions. This was found useful for time series BSS as diagonalizing many autocovariance matrices (SOBI) in comparison to diagonalize only one (AMUSE) reduces the influence of the chosen lag for the latter approach (Miettinen et al. 2012, 2016). A similar result was found for the spatial case by Bachoc et al. (2020). For the general use of joint diagonalization in multivariate statistics see also Nordhausen and Ruiz-Gazen (2022). Hence, joint diagonalization seems also reasonable to be considered in the context of stBSS; hence, it is referred to as spatio-temporal SOBI (stSOBI).

#### Definition 3.3

(stSOBI) Given a *p*-variate random process $\mathbf {x}(\mathbf {s}, t)$, which follows the stBSS model (Definition 2.1), let $\{f_1,\ldots ,f_L\}$ be a set of spatio-temporal kernel functions. Define the whitened version of $\mathbf {x}(\mathbf {s}, t)$ by ${\mathbf {x}}^{wh}({\mathbf {s}},t) = {\mathop {\mathbf {LACF}}\nolimits }_{f_0}^{-1/2}(\mathbf {x}(\mathbf {s}, t))(\mathbf {x}(\mathbf {s}, t)- {\mathbf {m}})$. Let ${{\mathbf {U}}}$ = ${{\mathbf {U}}}(\mathbf {x}(\mathbf {s}, t))$ be the orthogonal joint diagonalization matrix of $\mathop {\mathbf {LACF}}\nolimits _{f_l}(\mathbf {x}^{wh}({\mathbf {s}},t))$, of dimension $(p \times p)$, for $l = 1, \ldots , L$, maximizing

20 $$\begin{aligned} \sum _{l=1}^L\Vert diag({\mathbf {U}}^\top \mathop {\mathbf {LACF}}\nolimits _{f_l}(\mathbf {x}^{wh}({\mathbf {s}},t)) {\mathbf {U}}) \Vert ^2_F, \end{aligned}$$

where $diag(\cdot )$ is a diagonal matrix, whose diagonal elements are the diagonal entries of the matrix-valued argument, while $\Vert \cdot \Vert _F$ represents the Frobenius norm. The stSOBI unmixing matrix functional $\mathbf {W}= \mathbf {W}(\mathbf {x}(\mathbf {s}, t))$, based on joint diagonalization, equals $\mathbf {W}= {\mathbf {U}}^\top {\mathop {\mathbf {LACF}}\nolimits }_{f_0}^{-1/2}(\mathbf {x}(\mathbf {s}, t))$.

Exact joint diagonalization of more than two (positive semi-definite) matrices is only possible if all of them commute. At the population level, all involved local autocovariance matrices do commute but for a given sample this does not hold true due to the estimation error. Hence, algorithms that approximately jointly diagonalize all involved local autocovariance matrices need to be utilized. In the following, an algorithm based on Givens rotations, as outlined in Clarkson (1988); Cardoso (1989), is used. There exist also other options in the literature, see for example Illner et al. (2015).

It might be desirable to fix the order of the latent process in a way that it resembles spatio-temporal dependence (measured by the local autocovariance matrices) of the latent process in a decreasing manner. For stAMUSE this is achieved by ordering the diagonal elements of the diagonalized local autocovariance matrix. For stSOBI we define so-called pseudo-eigenvalues as

21 $$\begin{aligned} \lambda _{i,l} ^ 2 = ({\mathbf {u}}_i^\top \mathop {\mathbf {LACF}}\nolimits _{f_l}(\mathbf {x}^{wh}({\mathbf {s}},t)) \mathbf{u}_i)^2 \qquad \text {for} \qquad i=1,\dots ,p ~ \text {and} ~ l=1,\dots ,L. \end{aligned}$$

Here, ${\mathbf {u}}_i$ is the *i*-th column vector of the orthogonal joint diagonalizing matrix ${\mathbf {U}}$. Hence, the value $\lambda _{i,l}$ gives the spatio-temporal second order dependence for the *i*-th latent field component measured by the *l*-th spatio-temporal kernel. The order of the latent components might by determined by the order of $\sum _{l=1}^L \lambda _{i,l} ^ 2$, this criterion might be also used in a scree-plot setting to determine the most meaningful components and discard the ones with less spatio-temporal dependence.

The following Proposition states the identifiability condition and the affine equivariance property for stSOBI.

#### Proposition 3.2

For the stSOBI functional (Definition 3.3) the following two properties hold:

1. Identifiable iff there exists $l \in \{1,\dots ,L\}$ such that $(\mathop {\mathbf {LACF}}\nolimits _{f_l}(\mathbf {z}(\mathbf {s}, t)))_{ii} \ne (\mathop {\mathbf {LACF}}\nolimits _{f_l}(\mathbf {z}(\mathbf {s}, t)))_{jj}$, for all $i,j=1,\dots ,p$ and $i \ne j$, and
2. Affine equivariant.

The proof for Proposition 3.2 follows the same outline as (Matilainen et al. (2015), Lemma 1) or (Bachoc et al. (2020), Proposition 5). The identifiability condition of Proposition 3.2 is far less restrictive as the one seen in Proposition 3.1. Moreover, if stSOBI uses only one kernel, the method and identifiability condition reduce to stAMUSE. Thus, stSOBI might be seen as a generalization of stAMUSE.

### Relation to PCA

One of the most used multivariate statistical tool is PCA (Jolliffe 1986) which finds linear components of the multivariate dataset that maximize variance. This problem can be formulated in the present context as finding a matrix $\mathbf {W}_{PCA}$ that satisfies

22 $$\begin{aligned} \mathbf {W}_{PCA} \mathbf {W}_{PCA}^\top = \mathbf {I}_p \qquad \text {and} \qquad \mathbf {W}_{PCA} \mathop {\mathbf {LACF}}\nolimits _{f_0}(\mathbf {x}(\mathbf {s}, t)) \mathbf {W}_{PCA}^\top = \mathbf {D}. \end{aligned}$$

Here, the left equation reflects the orthogonality condition and the right equations states that the covariance matrix is diagonalized and $\mathbf {D}$ holds the variances of the found principal components on its diagonal. Interpretations of the results are usually achieved by the convenient loadings-scores scheme, where $\mathbf {W}_{PCA}$ is the matrix of loadings and $\mathbf {W}_{PCA}\mathbf {x}(\mathbf {s}, t)$ gives the scores. Note that PCA uses only the second moments of the marginal distributions which leads to the fact that it completely neglects the most important source of information for spatio-temporal data, namely spatio-temporal dependence. StBSS extends the former optimization equations by relaxing the orthogonal condition and using spatio-temporal dependence in terms of the $\mathop {\mathbf {LACF}}\nolimits$ matrices by

23 $$\begin{aligned} \mathbf {W}\mathop {\mathbf {LACF}}\nolimits _{f_0}(\mathbf {x}(\mathbf {s}, t)) \mathbf {W}^\top = \mathbf {I}_p ~ \text {and} ~ \mathbf {W}\mathop {\mathbf {LACF}}\nolimits _{f_l}(\mathbf {x}(\mathbf {s}, t)) \mathbf {W}^\top = \mathbf {D}_l ~ \text {for} ~ l = 1, \dots , L. \end{aligned}$$

The former equations also hint the advantages of stBSS. Firstly, the results can be interpreted in the same fashion as in PCA (loadings-scores principle) but the method specifically accounts for spatio-temporal second-order dependence. Secondly, the resulting latent process are not only uncorrelated marginally but also in their spatio-temporal dependence which leads to the fact that further analysis can be carried out on each entry of the latent process individually. This avoids the use of multivariate spatio-temporal statistics tools in favor of univariate ones.

In similar terms stBSS can also be seen as an extension of PCA when the whitening step is based on an eigenvalue decomposition of the covariance matrix by $\mathop {\mathbf {LACF}}\nolimits ^{-1/2}_{f_0}(\mathbf {x}(\mathbf {s}, t)) = \mathbf {V}\mathbf {D}\mathbf {V}^ \top$. Then, $\mathbf {W}_{PCA} = \mathbf {V}$ and the principal components, whitened observable and latent process are given by

24 $$\begin{aligned}&\mathbf {V}^\top (\mathbf {x}(\mathbf {s}, t)- \mathbb {E}(\mathbf {x}(\mathbf {s}, t))), \quad \mathbf {V}\mathbf {D}^{-1/2} \mathbf {V}^\top (\mathbf {x}(\mathbf {s}, t)- \mathbb {E}(\mathbf {x}(\mathbf {s}, t))) \quad \text {and} \end{aligned}$$

25 $$\begin{aligned}&{\mathbf {U}}^\top \mathbf {V}\mathbf {D}^{-1/2} \mathbf {V}^\top (\mathbf {x}(\mathbf {s}, t)- \mathbb {E}(\mathbf {x}(\mathbf {s}, t))). \end{aligned}$$

Thus, the latent process is a rescaled and rotated version of the principal components.

In practical considerations PCA and stBSS might be used in conjunction by firstly applying PCA as dimension reduction and then using stBSS on the retained principal components that show substantial variation. More information on the relation between PCA and BSS can be found in Nordhausen and Oja (2018).

## Simulations

In this part of the study the formerly introduced unmixing matrix functionals are validated on simulated datasets using the R program language version 3.6.1 (R Core Team 2021) with the help of the packages JADE (Miettinen et al. 2017), RandomFields (Schlather et al. 2015) SpatialBSS (Muehlmann et al. 2021a) and SpaceTimeBSS (Muehlmann et al. 2022b).

### Simulation settings

In order to simulate a multivariate spatio-temporal random field, the set of spatio-temporal locations has to be defined. The spatial domains are of the form $\mathcal {S}_{n_{sp}} = (0,n_{sp}] \times (0,n_{sp}]$ and the temporal domains are of the form $\mathcal {T}_{n_{t}} = [1, n_t]$ resulting in spatio-temporal domains $\mathcal {S}_{n_{sp}} \times \mathcal {T}_{n_{t}}$ which are abbreviated in the following by $[0,n_{sp}]^2 \times n_t$. For a given domain $[0,n_{sp}]^2 \times n_t$ the set of spatial sample locations is formed by overlaying the spatial domain with a grid $\mathcal {S}_{n_{sp}} \cap (\mathbb {Z}^2 / 4)$, then, $n_{sp}^2$ sample locations are randomly drawn for each simulation iteration from this resulting grid in order to simulate irregular sample locations. The temporal locations are simply $\mathcal {T}\cap \mathbb {Z}$ where $n_t$ equals twice $n_{sp}^2$ to resemble imbalanced spatio-temporal datasets which often occur in practice.

**Fig. 1:**
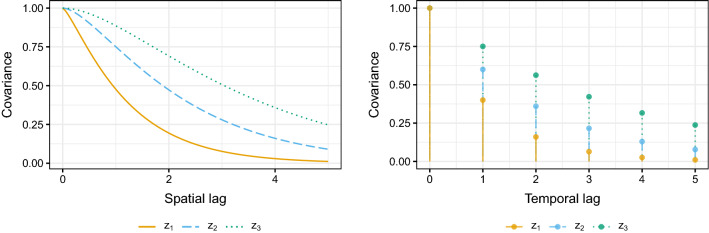
Covariance structure for the latent processes of Model 1 (left panel) and Model 2 (right panel). Model 1 consists of three stationary Matérn covariance functions with parameters $(\sigma ^2, \nu , \rho )$ equal to (1.0, 0.7, 1.0), (1.0, 1.0, 1.5) and (1.0, 1.3, 2.0) for $z_1, z_2$ and $z_3$. Model 2 consists of three stationary AR(1) covariance functions with parameters $(\sigma ^2, \phi )$ equal to (1.0, 0.4), (1.0, 0.6) and (1.0, 0.75) for $z_1, z_2$ and $z_3$

In the following simulation study, the latent fields follow the stBSS model (Definition 2.1) with $p = 3$, $\mathbf {m}= \mathbf {0}$ and $\mathbf {A}= \mathbf {I}_3$, which is without loss of generality due to the affine equivariance of the methods. The latent process is a centered Gaussian process where the spatio-temporal covariance follows one of the six following models.

Model 1 is a sole spatial model. For each time point a realization of a random field is independently sampled on the spatial locations. The entries of the random field are independent in time, while follow the well-known stationary Matérn covariance function (Guttorp and Gneiting 2006) in space, which is defined by

26 $$\begin{aligned} C^{mat}(h;\sigma ^2, \nu , \rho ) = \frac{\sigma ^ 2}{2 ^ {\nu - 1} \Gamma (\nu )} \left( \frac{h}{\rho } \right) ^ \nu K_\nu \left( \frac{h}{\rho } \right) . \end{aligned}$$

Here, $\sigma ^ 2 > 0$ is the variance, $\nu > 0$ is the shape and $\rho > 0$ is the scale parameter, *h* is the spatial lag, $\Gamma$ is the gamma function and $K_\nu$ is the modified Bessel function of second kind. The parameters $(\sigma ^2, \nu , \rho )$ equal (1.0, 0.7, 1.0), (1.0, 1.0, 1.5) and (1.0, 1.3, 2.0) for the latent processes $z_1$, $z_2$ and $z_3$, respectively. The left panel of Fig. 1 depicts the covariance functions for these parameter choices.

Model 2 is the opposite of Model 1 as for each spatial location a time series is independently sampled. The entries are independent in space and follow the exponential covariance structure resulting from an AR(1) process in time defined by

27 $$\begin{aligned} C^{exp}(\tau ; \sigma ^2, \phi ) = \sigma ^2 \phi ^ {|{\tau }|}. \end{aligned}$$

In the above form $\tau$ is the temporal lag, $\sigma ^ 2$ is the variance parameter and $\phi \in (-1, 1)$. The parameters $(\sigma ^2, \phi )$ for the latent processes $z_1$, $z_2$ and $z_3$ equal (1.0, 0.4), (1.0, 0.6) and (1.0, 0.75), respectively, which are depicted in the right panel of Fig. 1.

**Fig. 2:**
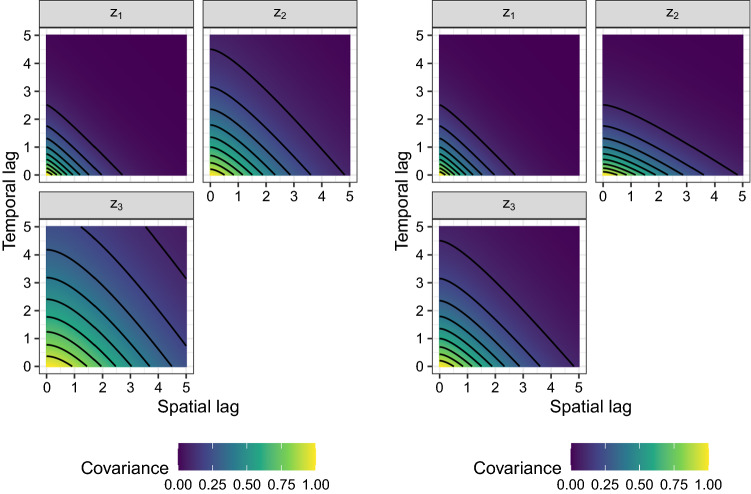
Covariance structure for the latent processes of Model 3 (left panel) and Model 4 (right panel). Model 3 and Model 4 are product models as seen in Equation (5) with $k_1 = 1$ and $k_2 = k_3 = 0$. The spatial part follows a Matérn covariance function and the temporal one an exponential functions. The parameters $\nu , \rho , \phi )$ equal (0.7, 1.0, 0.4), (1.0, 1.5, 0.6) and (1.3, 2.0, 0.75) for Model 3 and (0.7, 1.0, 0.4), (0.7, 1.0, 0.6) and (1.0, 1.5, 0.6) for Model 4

Model 3 & Model 4 are product models, obtained from Equation (5) with $k_1 = 1$ and $k_2 = k_3 = 0$. Both models use Matérn covariance functions for the spatial and exponential covariance functions for the temporal part (i.e.: $C^{sp}(h) = C^{mat}(h; 1, \nu , \rho )$ and $C^{t}(\tau ) = C^{exp}(\tau ; 1, \phi )$) where the parameters $(\nu , \rho )$ equal the ones of Model 1 and $\phi$ the ones of Model 2 for Model 3. Model 4 uses $(\nu , \rho )$ to be (0.7, 1.0), (0.7, 1.0) and (1.0, 1.5) for the spatial part of $z_1,z_2,z_3$, respectively and $\phi$ equals 0.4, 0.6, 0.6 for temporal part of the three latent random processes $z_1,z_2,z_3$, respectively. The resulting covariance surfaces are illustrated in Fig. 2.

**Fig. 3:**
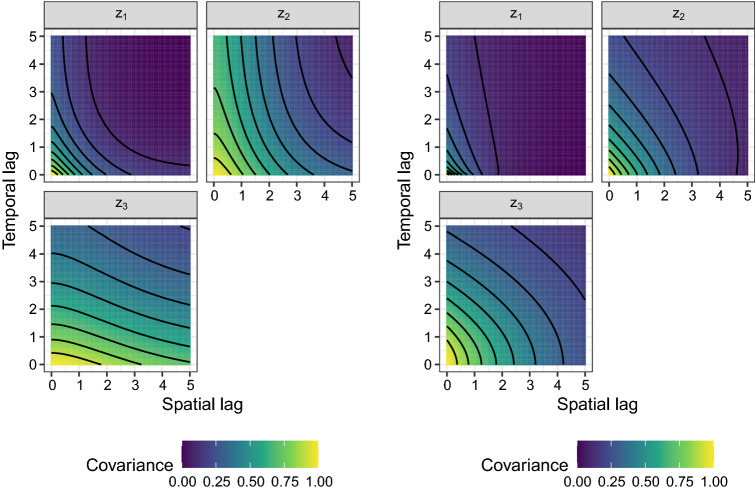
Covariance surfaces for the three entries of the latent processes for Model 5 (left panel) and Model 6 (right panel)

Model 5 is a product sum model (Equation (5)) with spatial Matérn and temporal exponential covariance functions. The parameters $(k_1, k_2, k_3, \nu , \rho , \phi )$ are equal to (5/8, 2/8, 1/8, 0.7, 1.0, 0.4), (1/8, 5/8, 2/8, 1.0, 1.5, 0.6) and (2/8, 1/8, 5/8, 1.3, 2.0, 0.75) for $z_1,z_2,z_3$, respectively. The covariance surfaces are seen in the left panel of Fig. 3.

Model 6 follows a Gneiting covariance function (Equation (6)) with $\psi (u) = u ^ {\alpha } / r_1 + 1$ and $\phi (h) = \exp {(- h ^{\gamma } / r_2)}$. These two functions combined with the general Gneiting form (Eq. (6)), with $d=2$ and $\sigma ^2=1$, leads to

28 $$\begin{aligned} C(h, \tau ) = \frac{ \sigma ^2 }{(u ^ {2 \alpha } / r_1 + 1) } \exp \left( - \frac{ h ^{2 \gamma }}{r_2 (u ^ {2 \alpha } / r_1 + 1) ^ {\gamma }} \right) , \end{aligned}$$

with the free parameters $0 < \alpha ,\gamma \le 1$, $r_1 > 0$ and $r_2 > 0$ collected in $(\alpha ,\gamma , r_1, r_2)$. These parameters are chosen to equal (0.35, 0.5, 0.85, 0.8), (0.6, 0.5, 2.3, 2.0) and (0.9, 0.5, 9.5, 3.5) for the entries $z_1$, $z_2$ and $z_3$ of the latent process. The right panel of Fig. 3 depicts the corresponding covariance surfaces.

For each simulated dataset an estimate of the unmixing matrix $\hat{\mathbf {W}}$ is computed by some BSS method as detailed in the subsequent sections. $\hat{\mathbf {W}}$ can be expected to fulfill $\hat{\mathbf {W}} \mathbf {A} \approx \mathbf {I}_3$ up to the model indeterminacy of order and sign. Note that this will be always the case for affine equivariant unmixing matrix functionals independently of the mixing matrix, therefore, the choice $\mathbf {A}= \mathbf {I}_3$ comes without loss of generality. Based on these considerations a performance index can be build around $\hat{\mathbf {W}} \mathbf {A} - \mathbf {I}_3$. One option is the so-called Minimum Distance Index (MDI) (Ilmonen et al. 2010; Lietzen et al. 2020) which is defined as follows

29 $$\begin{aligned} \text {MDI}(\hat{\mathbf {W}}, \mathbf {A}) = \frac{1}{\sqrt{p-1}} \inf _{\mathbf {C} \in \mathcal {C}^p} \Vert \mathbf {C} \hat{\mathbf {W}} \mathbf {A} - \mathbf {I}_p \Vert _F . \end{aligned}$$

Here, $\Vert \cdot \Vert _F$ is the Frobenius matrix norm and $\mathcal {C}^p$ is the set of $(p \times p)$ matrices with exactly one non-zero element in each row an column. The MDI takes values between zero and one where zero is achieved when $\hat{\mathbf {W}}$ equals $\mathbf {A}$ (up to sign and order). Note that there are many alternative performance measures as reviewed in Nordhausen et al. (2011) but the popular MDI index has been preferred as it fulfills all necessary conditions and has in addition many other nice properties like for example a direct connection to the limiting covariance matrix of the unmixing matrix estimate.

### Performance of different kernel settings

For each simulated dataset the unmixing matrix is estimated by stAMUSE and stSOBI where the kernel setting either considers sole spatial, sole temporal or spatio-temporal dependence. For only temporal dependence, a ball kernel with $\mathbf{h}=\mathbf{0}$ is used in conjunction with either $r_t=1$ denoted as stAMUSE.t or $r_t=1,2,3$ denoted as stSOBI.t. The sole spatial setting uses always $\tau = 0$ with one ring kernel with parameters (0, 1) denoted as stAMUSE.s or three ring kernels with parameters (0, 1), (1, 2), (2, 3) denoted as stSOBI.s. For the spatio-temporal kernels three settings are considered: $r_t = 1$ with a ring kernel with parameters (0, 1) denoted as stAMUSE.st, all nine kernel combinations originating from $r_t=1,2,3$ and ring kernels with parameters (0, 1), (1, 2), (2, 3) denoted as stSOBI.st and lastly all twelve kernels from the former stSOBI.t, stSOBI.s and stSOBI.st settings denoted as stSOBI.st2. In total stAMUSE.t, stSOBI.t, stAMUSE.s, stSOBI.s use only marginal spatial or temporal dependence, stAMUSE.st and stSOBI.st use spatio-temporal dependence, and stSOBI.st2 uses marginal spatial, marginal temporal and spatio-temporal dependence all with either simultaneous or joint diagonalization.

**Fig. 4:**
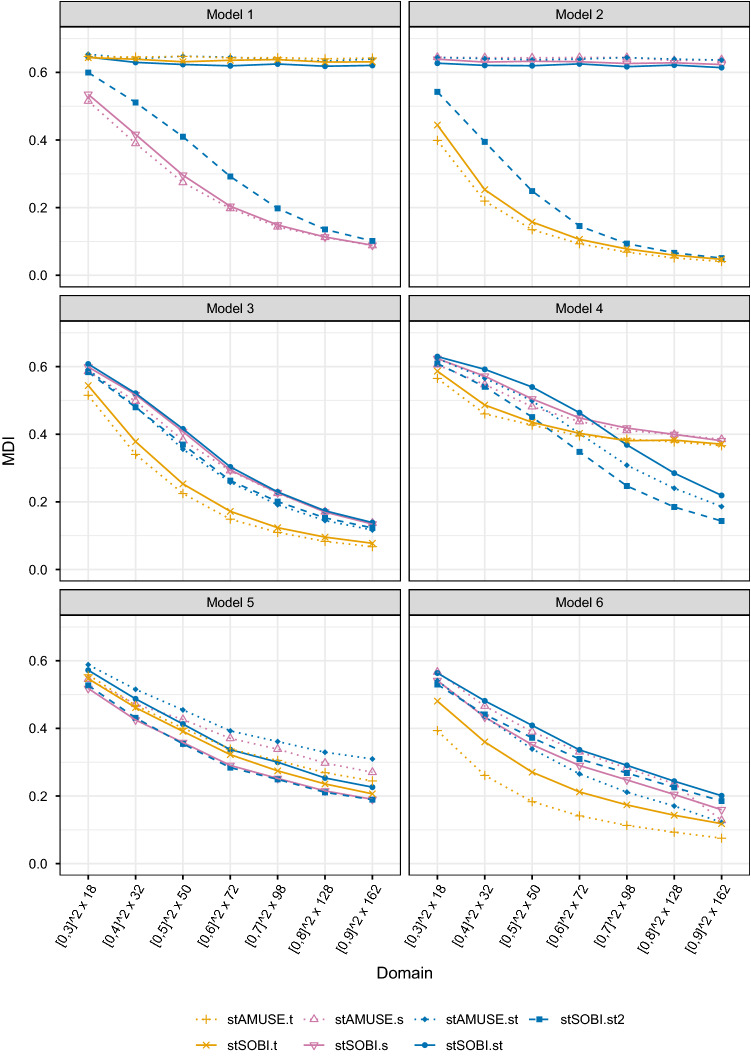
Average MDI computed based on 2000 simulation repetitions for different sample sizes, different stBSS methods and Models 1–6

The average MDI computed for 2000 simulation repetitions is depicted in Fig. 4 for the former estimators for Model 1 - 6 and different sample sizes. The obtained results are summarized below:

- Model 1 only exhibits spatial dependence which leads to the result that only kernel settings which utilize spatial dependence (stAMUSE.s, stSOBI.s and stSOBI.st2) deliver meaningful results. The circumstance that stSOBI.st2 performs slightly worse than the other two methods might be explained by the fact that stSOBI.st2 uses a total of twelve kernels where only three of them are sole spatial, hence, the other nine are non-informative ones only adding noise to the joint diagonalization algorithm.
- Equal quantitative results are seen for Model 2 where the role of space and time is exchanged.
- Model 3 is a product model that exhibits full spatio-temporal dependence, hence, all methods seem to work. However, in this model stAMUSE and stSOBI with sole temporal kernel settings show the best performance. An intuitive explanation might be given by the fact that for each dataset the number of times is double the number of spatial sample locations leading to less estimation error for a sole temporal kernel. Similarly, the exponential decaying temporal covariance functions yields that most of the information for signal separation is in the first lag, thus, stAMUSE.t shows the best performance for this model.
- Model 4 is special in the sense that the marginal spatial dependence is the same for the first and second entry of the latent field and the marginal temporal dependence is equal for the second and third entry of the latent process. For such a model only kernel settings that use full spatio-temporal dependence meet the required identifiability condition. Indeed, only the methods utilizing full spatio-temporal dependence are able to separate the signals for Model 4.
- Similarly as for Model 3, Model 5 and 6 show different marginal spatial and temporal as well as different full spatio-temporal dependence for all entries of the latent field. Thus, all methods are able to separate the signal and show very similar performance. For Model 6 the methods only utilizing temporal dependence again work best.

### Comparison to contender methods

This part of the simulations is devoted to comparing the stBSS methods with contender BSS methods and a random guess. The random guess is carried out by firstly whitening the data and then drawing an orthogonal matrix randomly. This procedure is motivated by the fact that all considered BSS methods share the whitening step but differ in the way of finding an orthogonal transformation, hence, whitening and a random orthogonal transformation acts as the worst possible method in the BSS context. For the contender BSS methods, the fourth order blind identification (FOBI) method (Cardoso 1989; Nordhausen and Virta 2019) has been used; this is designed for iid data and uses higher order moment for the signal separation. Moreover, the non-stationary source separation time delayed joint diagonalization (NSS.TDJ) method (Choi and Cichocki 2000) with $r_t = 1$ and the spatial non-stationary source separation spatial joint diagonalization (SNSS.SJD) method (Muehlmann et al. 2022a) with a ring kernel with parameters (0, 1) have been applied. The latter two methods are temporal and spatial non-stationary methods. NSS.TDJ diagonalizes autocovariance matrices for the time series observed for each spatial location, and SNSS.SJD diagonalizes local covariance matrices for the random fields observed for each time point.

**Fig. 5:**
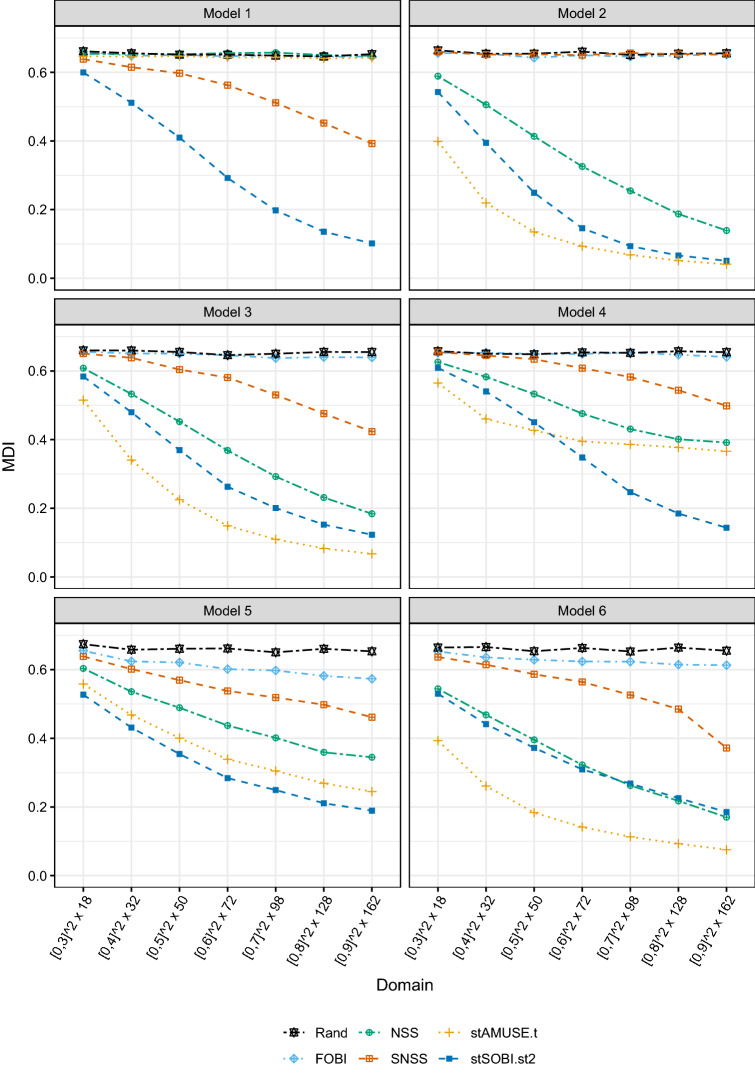
Average MDI computed based on 2000 simulation repetitions for different sample sizes, contender methods, stBSS methods and a random guess for Models 1–6

Figure 5 depicts the average MDI based on 2000 simulation repetitions for the three contender methods, stAMUSE.t, stSOBI.st2 and the random guess for Model 1–6 and different sample sizes. It is worth pointing out the following aspects:

- The quantitative results are similar for Model 1 and Model 2 as before. For Model 1 only SNSS and for Model 2 only NSS are able to separate the signals. The worse performance of SNSS in Model 1 compared to the performance of NSS for Model 2 might be again explained by the imbalance between information available in space and time for the datasets. For a given sample size NSS can use double the amount of samples to estimate the autocovariance compared to SNSS.
- NSS and SNSS are able to estimate the signal in Model 3, 5 and 6, where SNSS is always outperformed by NSS (intuitively explained again by the imbalanced datasets).
- Interestingly, NSS is able to perform equally wells as stSOBI in Model 6, since the temporal part of the sources is much more discriminant.
- In Model 4 the contender methods only use marginal spatial or temporal dependence, hence, they are not able to fully recover the signal. However, with increasing sample size these two contender methods are able to properly estimate two out of three latent process entries as the entries one and two have different marginal temporal and entries two and three have different marginal spatial dependence. This might explain their increasing performance with sample size.

## Data example

**Fig. 6:**
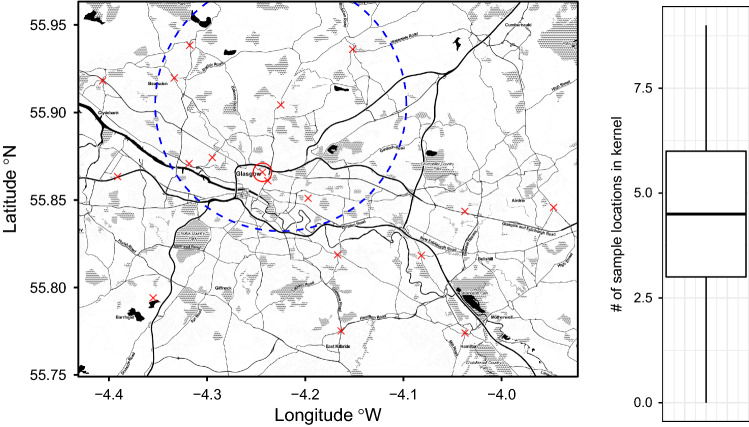
(Left) Map of Glasgow with the 18 considered measuring stations indicated by the red crosses. The blue circle indicates the used kernel parameter of 8000 m. Distances are computed in UTM zone 30. (Right) Boxplot depicting the number of neighbouring sample locations defined by the ring kernel for each sample location. Map tiles by Stamen Design, under CC BY 3.0. Data by OpenStreetMap, under ODbL

The aim of this section is to show how the proposed methods can be used in the analysis of a spatio-temporal dataset; moreover, possible ways of downstream analysis based on the stBSS results are presented. The considered datasets are hourly averaged measurements of nitric oxide (*NO*), nitrogen dioxide ($NO_2$) and particulate matter with a diameter not greater than 10 $\mu m$ ($PM_{10}$) in $\mu g /m^3$ at 18 different monitoring stations in the Glasgow area between 01/01/2017 and 12/31/2017. This dataset is provided by air quality monitoring sites operated by the Scottish Government and Local Authorities from http://www.scottishairquality.scot/data/. The left panel of Fig. 6 depicts the considered sample locations. The measurements show a diurnal behaviour which was removed by using the algorithm REMOVEMULT (De Iaco et al. 2010) prior the following analysis. In total, the deseasonalized dataset is three-variate and contains 130894 spatio-temporal samples. Note that for each space-time location each of the three measurements are available except for some missing  values which are assumed to occur  randomly. This is not a problem in estimating the local autocovariance matrices as the specific time of the measurements are accounted for when computing lags amongst them.

**Table 1:**
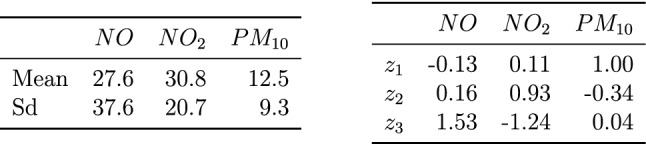
Sample mean and sample standard deviation for each original variable (left). Loadings matrix (unmixing matrix) for the three found latent spatio-temporal random fields (right)

Besides the kernel setting, hereafter discussed, also the scale of the original data must be considered before applying stBSS. If the scale differs between variables the loadings are difficult to compare. The left Table 1 shows the sample mean and sample standard deviation of the original data. As the scales are different in one order of magnitude, the data have been scaled to zero mean and unit variance before applying stBSS. Note that as the transformation is affine, it does not affect the resulting latent components as stBSS methods are affine equivariant. Moreover as clarified, in  a first step of stSOBI proper spatio-temporal kernels need to be chosen. As this dataset is highly imbalanced between space and time it might be a good strategy to focus on serial kernels and only consider a few spatial ones. For the spatial parts of the kernel, the radius equals 8000 m in order to include enough neighbouring samples per spatial sample location as seen in the boxplot on the right panel of Fig. 6. In the left panel of Fig. 6 the blue circle depicts this parameter choice. For the temporal lags, the lags between one and ten hours are included and also the lags 12 and 24 hours. The latter two choices are meant to account for effects from morning and evening traffic. In total, 16 kernels have been considered, where one is sole spatial (ring kernel with serial lag zero and spatial parameters $(r_{s_0},r_{s_1})= (0,8000)$ m), three spatio-temporal (ring kernel with serial lags 1, 2 and 3 and spatial parameters $(r_{s_0},r_{s_1})= (0,8000)$ m) and twelve sole temporal (ball kernel with lags 1 to 10, 12 and 24 and spatial parameter $r_s = 0$ m). Table  2 summarizes these choices. Generally, the choice of the considered kernel settings should be based on domain experts as the most informative temporal and spatial lags might be identified by specific domain knowledge. Another strategy might be given by simply trying out different kernel settings and use the ones which produce stable solutions in terms of the unmixing matrix and the found components. This strategy is based on the fact that if the stBSS model holds, the solution is independent of the kernel setting (for all kernel settings that satisfy the identifiability conditions). Thus, stable solutions should indicate valid and useful kernel settings.

**Table 2:** Pseudo-eigenvalues for the 16 used kernel functions. Type is the type of the kernel. Par gives used parameters for the spatial part in meters. Lag is the used lag in hours

Type	Par	Lag	$z_1$	$z_2$	$z_3$	Type	Par	Lag	$z_1$	$z_2$	$z_3$
Ring	(0, 8000)	0	1.39	1.25	1.08	Ball	0	5	0.84	0.88	0.57
Ring	(0, 8000)	1	1.91	1.71	1.41	Ball	0	6	0.78	0.83	0.51
Ring	(0, 8000)	2	1.79	1.58	1.18	Ball	0	7	0.73	0.79	0.46
Ring	(0, 8000)	3	1.67	1.45	0.94	Ball	0	8	0.69	0.76	0.43
Ball	0	1	1.21	1.26	1.19	Ball	0	9	0.65	0.73	0.40
Ball	0	2	1.09	1.14	0.99	Ball	0	10	0.62	0.71	0.37
Ball	0	3	0.99	1.03	0.81	Ball	0	12	0.57	0.66	0.34
Ball	0	4	0.91	0.94	0.67	Ball	0	24	0.48	0.68	0.35

After running stSOBI the pseudo-eigenvalues, unmixing matrix and found processes can be used for interpretation and further analysis. As the found transformation is linear, the unmixing matrix can be seen as the loadings matrix and the found processes are the scores leading to the same interpretation scheme as in classical PCA. The right Table 1 shows the loadings matrix and Table 2 summarizes the pseudo-eigenvalues for the used 16 local autcovariance matrices. The pseudo-eigenvalues give an idea which kernel setting is important for which latent process. E.g.: higher values for the serial kernels for lags 12 and 24 of $z_2$ indicate that this lags are more important than for $z_1$ and $z_3$; similarly, higher values for the spatial and spatio-temporal kernels of $z_1$ indicate that the chosen lags are more significant than for $z_2$ and $z_3$. The sums of the columns can also be used in scree-plot setting, however, for this three-variate dataset the pseudo-eigenvalues show that all three components are important. For a similar use of such pseudo-eigenvalues in other BSS contexts see also Matilainen et al. (2019); Muehlmann et al. (2021c).

The loadings (right Table 1) indicate that $z_1$ is mainly driven by $PM_{10}$ and the difference between *NO* and $NO_2$. Similarly, $z_2$ is formed mainly by $NO_2$ and roughly the difference of *NO* and twice $PM_{10}$. Lastly, $z_3$ could be seen as the difference between *NO* and $NO_2$ with a negligible effect of $PM_{10}$. This difference represents the discrepancy between primary and secondary pollutants, as *NO* and $NO_2$, respectively. In the event of accidental pollution by nitrogen monoxide, the concentration decays in 2-5 days, but in the case of continuous emissions (for example in urban areas with heavy vehicular traffic), the activation of a daily cycle leads to the production of secondary pollutants, such as nitrogen dioxide.

**Fig. 7:**
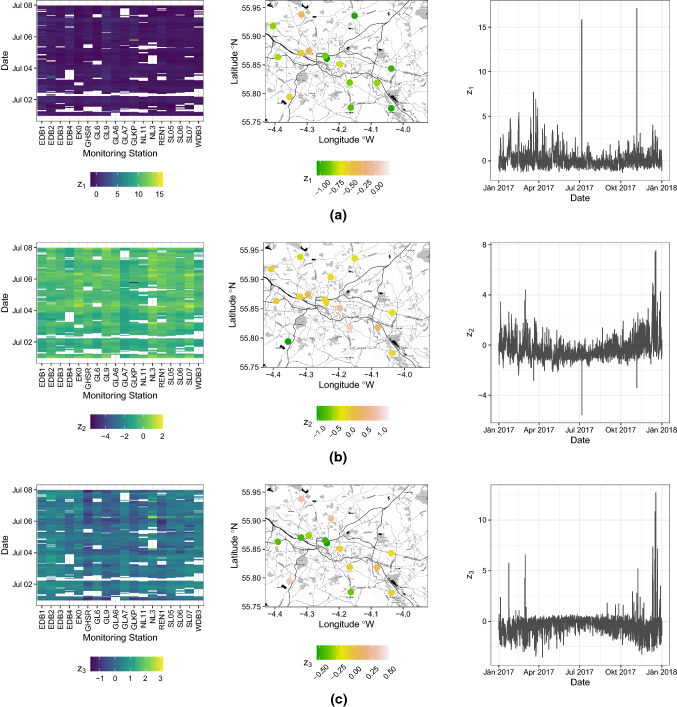
Hovmöller diagram for the first week of July 2017 for all 18 stations (left). Map of the 18 stations for 07/01/2017 (middle). Time series plot for the station indicated by the red circle in Fig. 6 (right), for: a) $z_1$, b) $z_2$, c) $z_3$ Map tiles by Stamen Design, under CC BY 3.0. Data by OpenStreetMap, under ODbL

Another advantage of stBSS is the fact that the found latent processes are marginally and spatio-temporally uncorrelated. Thus, in a downstream analysis each process can be considered individually. For example, visual tools such as Hovmöller diagrams (Allard et al. 2017) for certain time periods and monitoring stations, spatial maps for different time points and time series plots for certain monitoring sites can be used to further investigate the latent processes. Hence, visualization of cross-dependencies are discarded. Figure 7(a–c) show these tools for the three latent processes.

Further interpretations of the results can be carried out with these plots in conjunction with the loadings matrix. E.g.: The time series plot for one station seen in the right panel of Fig. 7(c) indicates that $z_3$ is higher in absolute value, during winter months and lower during summer, spring and autumn. As $z_3$ is mainly driven by the difference of *NO* and $NO_2$ this means that the concentration of $NO_2$ is higher compared to *NO*, the opposite happens during the other months of the year. A similar effect is also seen in the time series plot of Fig. 7(b) right panel for $z_2$.

**Fig. 8:**
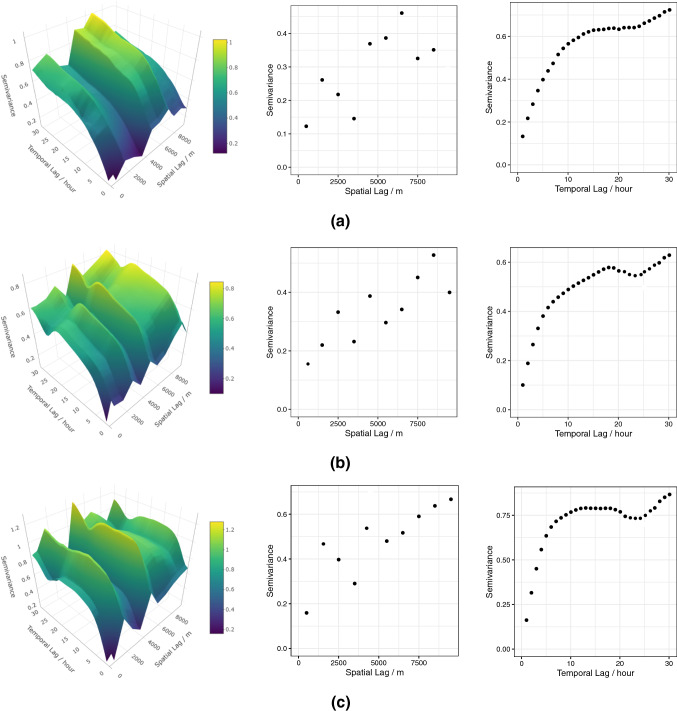
Variogram surface (left), marginal spatial (middle) and marginal temporal (right) variogram for the latent random field a) $z_1$, b) $z_2$, c) $z_3$

In spatio-temporal prediction the uncorrelatedness property of the latent realizations can be utilized advantageously as well. As underlined in Muehlmann et al. (2021b), this can be done by using univariate prediction tools for each component of the latent process individually in order to predict each regionalized variable at the desired spatio-temporal location. E.g.: univariate spatio-temporal Kriging can be used based on structural analysis conducted separately for each latent random field. For the specific case study, the three sample covariance functions or variograms for $z_1$, $z_2$ and $z_3$, as seen in Fig. 8 have to be computed, then on the basis of the fitted models, three individual Kriging predictions for the three sources can be collected into a vector and then multiplied by the inverse of the loadings matrix which forms the prediction of the original data at the unobserved spatio-temporal location. This reduces computation time and model complexity significantly, as no cross-dependencies need to be estimated and modeled. Especially in a space-time domain, modeling direct and cross covariance functions is burdened by the evaluation of the corresponding three-dimensional empirical covariance surfaces, from which is not easy to stem reasonable assumptions on the multivariate random field (such as the number of variability scales or the presence of anthropic factors influencing the phenomenon). On the other hand, univariate modeling of spatio-temporal covariance functions can be supported by the existence of numerous classes of space-time covariance functions which are able to cover a wide spectrum of dependence structures; moreover, some computational tools can also help users to select the appropriate class for the given spatio-temporal sample covariance surface.

## Conclusions

In this contribution the BSS methods for data that exhibit spatio-temporal dependence were presented. A new scatter functional, namely local autocovariance matrices, which characterizes spatio-temporal second-order dependence, was introduced. A discussion on different forms of spatio-temporal kernel functions, based on different possible metrics, was proposed. Two novel methods (stAMUSE and stSOBI) which simultaneously or jointly diagonalize these scatter matrices to recover spatio-temporally uncorrelated sources were also introduced. The simulation study confirmed the usefulness of these methods. It was also emphasized that the stBSS methodology can be seen as a special case of ST-LMC, and as such it acts also as a way of modelling multivariate spatio-temporal covariance. But the real strength of the proposed methodology lies in the fact that the found signals are spatio-temporally uncorrelated. This in turn means that downstream analysis of the original multivariate date can be carried out with univariate statistical tools on each component of the sources individually. Hence, pre-processing the data with an stBSS method reduces multivariate modelling to several univariate ones, modelling of cross-dependencies is discarded.

So far, a model based on second-order stationary spatio-temporal random fields, was considered. Obviously, these assumptions are highly likely to be not fulfilled in the two following ways. Either a trend might be present in the data and/or the second order dependence specifically depends on space-time locations. For the latter case a first adaptation of the stBSS methods can be given by assuming local stationarity and computing local autocovariance matrices for parts of the spatio-temporal domains. The influence of a trend can be reduced by replacing the local autocovariances with scatters that are based on difference processes. Such a procedure is common practice in geostatistics where often the variogram is favoured over the covariance. Characterizing the large sample behaviour of local autocovariance matrices and the unmixing matrix estimators is desirable in order to build asymptotic tests and provide estimation errors. Furthermore, to aid practitioners in choosing reasonable kernel settings, visual analytic tools which were developed for SBSS, AMUSE and SOBI in Piccolotto et al. (2022a, 2022b) will be extended to the spatio-temporal case.

The choice of suiting kernel functions and their parameters is an open question which is generally considered a challenging task in BSS (Tang et al. 2005). The problem lies in the fact that the quality of the separation  depends on the dissimilarity of the eigenvalues of the matrices $\mathop {\mathbf {LACF}}\nolimits _{f}(\mathbf {z}(\mathbf {s}, t))$ (see Propositions 3.1 and 3.2). These eigenvalues are not accessible as the latent random field is unknown a-priori. Therefore, it is advisable to rather use stSOBI with a larger number of kernels. A very practical guideline might be given by simply using equidistant ring kernels to a maximum extend of half the maximum distance (similarly as in variogram estimation) for the spatial part and a small number of lags for the temporal part. Note, that for a very imbalanced dataset it might be a good strategy to use kernels which account for the imbalance. For example, if the temporal resolution is much higher compared to the spatial, a small number of spatial but a higher number of temporal kernels might be suitable. Besides these insights, it is also possible to estimate the latent field with a high number of kernels and sort out uninformative ones based on asymptotic arguments. Such a strategy is utilized for temporal BSS by Taskinen et al. (2016).

## Data Availability

Data can be found at the link http://www.scottishairquality.scot/data/. Implementations of stAMUSE and stSOBI are made available in the R package SpaceTimeBSS which can be downloaded from CRAN.
